# Developmental Exposure to Perchlorate Alters Synaptic Transmission in Hippocampus of the Adult Rat

**DOI:** 10.1289/ehp.11089

**Published:** 2008-03-06

**Authors:** Mary E. Gilbert, Li Sui

**Affiliations:** 1 Neurotoxicology Division, U.S. Environmental Protection Agency, Research Triangle Park, North Carolina, USA; 2 Department of Psychology, University of North Carolina at Chapel Hill, Chapel Hill, North Carolina, USA; 3 National Research Council, Washington, DC, USA

**Keywords:** brain, cognition, development, hippocampus, iodine, learning and memory, neurotoxicity, perchlorate, thyroid hormone

## Abstract

**Background:**

Perchlorate is an environmental contaminant that blocks iodine uptake into the thyroid gland and reduces thyroid hormones. This action of perchlorate raises significant concern over its effects on brain development.

**Objectives:**

The purpose of this study was to evaluate neurologic function in rats after developmental exposure to perchlorate.

**Methods:**

Pregnant rats were exposed to 0, 30, 300, or 1,000 ppm perchlorate in drinking water from gestational day 6 until weaning. Adult male offspring were evaluated on a series of behavioral tasks and neurophysiologic measures of synaptic function in the hippocampus.

**Results:**

At the highest perchlorate dose, triiodothyronine (T_3_) and thyroxine (T_4_) were reduced in pups on postnatal day 21. T_4_ in dams was reduced relative to controls by 16%, 28%, and 60% in the 30-, 300-, and 1,000-ppm dose groups, respectively. Reductions in T_4_ were associated with increases in thyroid-stimulating hormone in the high-dose group. No changes were seen in serum T_3_. Perchlorate did not impair motor activity, spatial learning, or fear conditioning. However, significant reductions in baseline synaptic transmission were observed in hippocampal field potentials at all dose levels. Reductions in inhibitory function were evident at 300 and 1,000 ppm, and augmentations in long-term potentiation were observed in the population spike measure at the highest dose.

**Conclusions:**

Dose-dependent deficits in hippocampal synaptic function were detectable with relatively minor perturbations of the thyroid axis, indicative of an irreversible impairment in synaptic transmission in response to developmental exposure to perchlorate.

Thyroid hormones play crucial roles in the development and maturation of the central nervous system. Severe reductions during critical periods in the prenatal and early postnatal period produce stunted growth and mental retardation in children (for review, see Anderson et al. 2003; Bernal 2002). However, recent reports indicate that children born to women experiencing modest subclinical perturbations of the thyroid axis during pregnancy have reduced IQ (intelligence quotient) scores and subtle deficits in cognition, memory, and visuospatial ability and a higher incidence of attention deficit hyperactivity disorder (ADHD) (Haddow et al. 1999; Zoeller and Rovet 2004). Neuroanatomic alterations have also been demonstrated in animal models of modest thyroid dysfunction in early development (Auso et al. 2004; Goodman and Gilbert 2007; Lavado-Autric et al. 2003). These structural deficits are accompanied by impairments in synaptic transmission, auditory function, behavioral and neurophysiologic assessments of learning and memory, and increased seizure sensitivity (Auso et al. 2004; Gilbert et al. 2006; Gilbert and Sui 2006; Goldey et al. 1995; Lavado-Autrec et al. 2003; Sui and Gilbert 2003).

Perchlorate is an environmental contaminant that reduces thyroid hormone (Wolff 1998). Ammonium perchlorate is a salt used primarily in solid rocket fuel and propellants, explosives, pyrotechnics, and blasting formulations. Improper disposal and use result in release of the salt to the environment, where it rapidly dissociates to perchlorate anion. Perchlorate anion has been detected in drinking water supplies, fruits, vegetables, grain, and dairy products [U.S. Environmental Protection Agency (EPA) 2002]. Perchlorate blocks the uptake of iodide, an element critical for thyroid hormone synthesis, by competitive inhibition at the sodium-iodine symporter (NIS) in the thyroid gland (Wolff 1998). This action is sufficient to reduce circulating levels of thyroid hormone and may induce neurotoxicity in developing organisms (U.S. EPA 2002; York et al. 2005a). Dohan et al. (2007) recently demonstrated that the NIS transports perchlorate with a higher affinity than it does iodine, and perchlorate accumulates in breast milk. Therefore, breast-fed infants may be subjected to higher concentrations of perchlorate than previously thought, compromising hormone production by reducing concentration of maternal iodine in milk in addition to directly inhibiting thyroidal uptake of iodine.

Recent data from the Centers for Disease Control and Prevention (CDC) National Health and Nutrition Survey reveal a strong association between perchlorate exposure in women in the general U.S. population and circulating levels of thyroid hormone (Blount et al. 2006; Steinmaus et al. 2007). Evidence of neurodevelopmental sequelae in clinical studies and in animal models with low-level thyroid disruption and recent observations of the extent of perchlorate exposure in the general U.S. population have raised considerable public health concerns over the effects of low-level perchlorate ingestion (Ginsberg and Rice 2005; Ginsberg et al. 2007; U.S. EPA 2002).

The hippocampus is a brain structure necessary for some types of learning and memory, and its structural integrity is dependent upon adequate supplies of thyroid hormone during development (Auso et al. 2004; Madeira et al. 1991, 1992). The relationship between thyroid hormone insufficiencies during critical periods of hippocampal development and ensuing functional deficits has not been defined. Although structural and functional alterations in hippocampus are profound with severe thyroid hormone reductions (Akaike et al. 1991; Dong et al. 2005; Gilbert and Paczkowski 2003; Vara et al. 2002), recent findings indicate that severe hypothyroidism is not required for their induction. Alterations in the expression of thyroid hormone–responsive genes in hippocampus and cortex, errors in neuronal migration of both excitatory and inhibitory neurons, changes in phenotypic expression of interneurons, and aberrant glial cell fate determinations have recently been demonstrated with modest degrees and brief episodes of thyroid hormone insufficiency (Auso et al. 2004; Gilbert et al. 2006; Goodman and Gilbert 2007; Lavado-Autric et al. 2003; Sharlin et al. 2008).

We designed the present study to examine the impact of perturbations of the thyroid axis associated with developmental exposure to perchlorate, using electrophysiologic and behavioral assessments of the hippocampus in rodents. Perchlorate induced a disruption of hormonal status in the neonate that recovered upon termination of exposure, yet a pronounced deficit in neurophysiologic properties of the hippocampus was evident in adulthood. These findings indicate that neurologic impairment is associated with modest degrees of thyroid hormone insufficiency and support previous animal studies of neurodevelopmental sequelae associated with low levels of perchlorate exposure (U.S. EPA 2002; York et al. 2005a).

## Methods

### Animal treatment

Pregnant Long–Evans (*n* = 106) rats were obtained from Charles River (Raleigh, NC) on gestational day (GD) 2 and housed individually in standard plastic hanging cages in an approved animal facility. All animal treatments were in strict accordance with the National Institutes of Health’s *Guide for the Care and Use of Laboratory Animals* (Institute of Laboratory Animal Resources 1996). All animals were treated humanely and with regard for alleviation of pain and suffering. Animal rooms were maintained on a 12:12-hr light:dark schedule, and animals were permitted free access to food (Purina 5001 rodent chow; Ralston-Purina, St. Louis, MO) and tap water. Beginning on GD6 and continuing until postnatal day (PND) 30, dams were administered 0, 30, 300, or 1,000 ppm ammonium perchlorate (ClO_4_; Sigma, St. Louis, MO) in the drinking water. The day of birth was designated PND0, and all litters were culled to 10 pups on PND4, equating as much as possible the number and pups of each sex in a given litter. Blood was collected from animals culled on PND4 and pooled within a litter regardless of sex, and hormone determinations were performed when sufficient quantities of serum were available. One male and one female pup per litter were sacrificed on PNDs 14 and 21 for determinations of circulating levels of thyroid hormones and for brain and hippocampal weights. On PND30 the offspring were weaned, transferred to plastic hanging cages (two to four per cage), and permitted free access to food and tap water.

Water consumption was monitored twice weekly during gestation and lactation. Dam weights were monitored frequently throughout pregnancy and lactation, and offspring weights were recorded during the first post-natal month and again in adulthood. Eye opening was examined by daily observation between PND15 and PND19, and the ratio of pups within a litter with both eyes open was determined. Behavioral and electrophysiologic tests were performed on adult male offspring as described below; only a single animal per litter is represented in any of the assessments.

### Hormone analysis

Trunk blood from pups on PNDs 4, 14, 21, and 80–90, and from dams at weaning on PND30 was collected after decapitation and allowed to clot on ice for a minimum of 30 min. Serum was separated via centrifugation of clotted samples and stored at −80°C for later analyses. We analyzed serum concentrations of total thyroxine (T_4_) and total triiodothyronine (T_3_) by radioimmunoassay (Diagnostic Products Corp., Los Angeles, CA), and thyroid-stimulating hormone (TSH) was measured using a standard double antibody assay as described by Thibodeaux et al. (2003). All samples were run in duplicate, and the intraassay and inter-assay variations ranged from 9 to 12%. We removed outliers from the data set if the value fell > SDs above or below the mean for the dose group. The lowest calibrator was 5 ng/mL for T_4_ assays and 10 ng/mL for T_3_ assays. The minimum detectable concentration (MDC) for each assay was determined statistically (3 SDs above background). The MDCs were 4.9 ng/mL for T_4_ assays and 7.8 ng/mL for T_3_ assays. In those cases where the sample result fell below the level of the lowest calibrator, the result was set by default to the MDC for statistical purposes.

### Behavioral assessments

We conducted three behavioral tasks—motor activity, spatial learning, and fear conditioning—in adult male offspring. See Supplemental Material available online (http://www.ehponline.org/members/2008/11089/suppl.pdf) for additional methodologic details.

### Motor activity

We assessed motor activity as a general test of neurotoxicity. Adult male offspring (*n* = 8–11 per group) were tested at 13 months of age using six photocell devices. Five successive 6-min intervals of activity were recorded during a single 30-min session. All testing occurred between 0700 and 1200 hours on the same day, and the order of testing was counterbalanced across dose groups.

### Morris water maze

We assessed spatial learning using the Morris water maze, as previously described (Gilbert and Sui 2006). At 3 months of age, male offspring (11–17 per dose group) were administered two daily trials (with 3–5 min between trials) for 15 consecutive days. The maze consisted of a water-filled circular tank located in a small room with salient and invariant visual cues posted on the walls. Four locations around the edge of the pool were defined as start points, and a circular escape platform was placed just below the surface of the water of one quadrant. Animals were placed into the tank and allowed to search for the escape platform for a maximum of 60 sec. Latency to reach the escape platform was recorded, and the animals were permitted to rest on it for 15 sec. A series of probe trials was conducted on trial 1 of test days 3, 6, 9, 12, and 15 in which the platform was removed and the animals were allowed to swim freely for 60 sec. The percentage of time the animals spent in each quadrant of the pool was recorded for each probe trial.

### Fear conditioning

We examined trace fear conditioning because it requires the integrity of the hippocampus (Bangasser et al. 2006). Conditioning was assessed at 7–8 months of age. We conducted testing in two chambers of identical dimensions, one for fear training and context testing, and a second, located in a different room, for cue testing. Conditioned stimulus (CS) and unconditioned stimulus (US) pairs were presented six times on day 1. A 30-sec trace interval separated the CS (15-sec compound light/tone stimulus) offset and the US (1 mA, 0.5-sec footshock through grid floor) onset, with a 3-min intertrial interval. The chamber was dark, and sound-attenuating chamber doors remained closed during training. The following day, context learning was assessed by returning animals to the training chamber and monitoring activity for 5 min via an infrared motion detector (Colbourn Instruments, Allentown, PA) mounted on the ceiling of the test chamber. Animals were returned to holding cages in a dimly lit room for 1 hr. Conditioning to cue was evaluated thereafter by placing animals in a test chamber located in a brightly lit room, chamber walls painted with black and white vertical stripes, with a smooth white Plexiglas floor, and sprayed with apple-scented disinfectant to provide a distinct olfactory cue. Activity was monitored for 2 min before and after six CS presentations separated by a 2-min intertrial interval.

### Electrophysiologic assessments. Surgical procedures

Adult male offspring (5–9 months of age) were anesthetized with urethane (1–2 g/kg by intraperitoneal injection) and prepared for stereotaxic surgery according to procedures described previously (Gilbert and Sui 2006). Two animals were assessed each day, and dose groups were counterbalanced over days to equate the mean age across groups. Data are presented for 16, 17, 16, and 14 animals for the 0-, 30-, 300-, and 1,000-ppm dose groups, respectively. Each animal was mounted in a stereotaxic frame, and a bipolar twisted stainless steel wire electrode was lowered into the angular bundle of the perforant path according to flat skull stereotaxic coordinates (from bregma, −7.2 mm posterior, 4.1 mm lateral). A monopolar insulated nichrome wire was lowered into the ipsilateral dentate gyrus (−3.5 mm posterior, 2.2 mm lateral) to record field potentials from the dentate gyrus. Nominal depths for stimulating and recording electrodes were 2.2 and 3.5 mm below dura, respectively, but optimal depth placement was achieved through electrophysiologic monitoring of the response evoked in the dentate gyrus after single-pulse perforant path stimulation.

The field potential comprises an initial positive component, the excitatory postsynaptic potential (EPSP), and a negative compound action potential, the population spike (PS). The positive component provides an index of synaptic activity comprising the summed EPSPs at the level of the dendrites (Lomo 1971). The slope of the EPSP was calculated as the rate of amplitude change for the initial positive component of the dentate gyrus field potential prior to PS onset. The negative component, PS, provides a measure of cell excitability, the number of granule cells firing action potentials in response to EPSP (Lomo 1971). PSs were estimated by the amplitude of a line connecting the lowest value of the negative potential to the point of intersection of a tangent connecting the two positive peaks of the potential.

### Input/output functions

Input-output (I/O) functions describe the relationship between the intensity of current applied to the perforant path (input = current) and the magnitude of the subsequent voltage change induced in the dentate gyrus (output = voltage).

Examination of I/O functions across a range of stimulus strengths characterizes the efficiency of excitatory synaptic transmission across this monosynaptic connection. A series of 25 intensities were delivered ranging from 20 to 1,500 μA (base to peak), 10 pulses/intensity (biphasic square wave pulses, 0.1-msec duration using a Grass S-88 stimulator and PSIU-6 constant current converters; both from Grass Technologies, West Warwick, RI), 10 sec between each pulse. Responses were amplified, digitized (33-kHz sampling rate), averaged using LabWindows (National Instruments, Austin, TX) and custom-designed software, and stored on a personal computer for later analysis. Both EPSP and PS measures were taken from these averaged responses to generate a curve characterizing the current–voltage relationships between perforant path stimulation and response amplitude across dose groups.

### Paired pulse depression and facilitation

Somatic output of cortical networks is modulated by local circuit interneurons, the majority of which have gamma amino butyric acid (GABA) as their neurotransmitter (Fukuda et al. 1996). An estimate of the integrity to somatic inhibition is derived by delivering pairs of stimulus pulses at various interpulse intervals (IPIs) and expressing the amplitude of PS evoked by the second pulse relative to the first (Burdette and Gilbert 1995). Paired pulse determinations of synaptic depression and enhancement were collected upon completion of baseline I/O functions in control and perchlorate-treated animals. Two pulses of equal strength were delivered (IPIs = 10, 20, 30, 70, and 250 ms) at three stimulus intensities. Intensities were chosen to produce PS amplitudes of the first pulse (conditioning pulse) equivalent to 20%, 50%, and 100% of the maximal PS amplitude recorded at 1,500 μA. Ten pulse pairs were averaged for each animal at each IPI and at each intensity. Data are expressed as a ratio of test pulse to conditioning pulse PS amplitude. A ratio < 1 reflects paired pulse depression, a ratio > 1 reflects paired pulse facilitation.

### Long-term potentiation

Hippocampal long-term potentiation (LTP) is a form of synaptic plasticity that has been studied intensively as a cellular model of learning. It has been evaluated *in vitro* in hippocampal area CA1 and in the dentate gyrus of the intact animal to identify molecular substrates and to link physiologic to behavioral attributes of learning (Bliss and Collingridge 1993; Martinez and Derrick 1996). LTP was assessed after the collection of paired pulse functions and the baseline I/O function. A probe stimulus (intensity producing a PS 50% of maximal) was delivered before and after train delivery to monitor the magnitude of evoked LTP. LTP was induced by delivering three train pairs (i.e., two 4-pulse bursts at 400 Hz with a 200-msec interval between each burst, repeated three times at 10-sec intervals) at a high stimulus strength (1,500 μA). Averaged responses were sampled at the probe stimulus intensity immediately and 15 min after train delivery. At completion of electro-physiologic testing, animals were sacrificed by decapitation and the brain was immersion fixed in 4% paraformaldehyde for histologic verification of electrode placement.

### Statistical analyses

All statistical analyses were conducted using SAS, version 6.12 (SAS Institute Inc., Cary, NC). We evaluated dam and pup body weights, with litter as the unit of analysis, using repeated-measures analysis of variance (ANOVA) with between-subjects effects of dose and day and a within-litter effect of sex. Sufficient serum and samples from each sex from each litter were not always available, so hormone data were first evaluated using ANOVA for the main effects of dose and sex, and dose × sex interactions at each age. As no main or interaction effects of sex were observed for any hormone estimates, the mean value of male and female pups from each litter was calculated and formed the data for subsequent analyses. These data were then subjected to ANOVA using litter as the unit of analysis. Where significant effects were found in hormone measures, mean contrast tests were conducted using Dunnett’s *t*-test (1-tailed tests, α = 0.05) to compare each dose group with control.

Repeated-measures ANOVA was conducted for motor activity, water maze learning, and fear conditioning to cue. One-way ANOVA was used to assess context conditioning and hippocampal LTP. Differences in baseline synaptic transmission were evaluated by subjecting EPSP slope and PS amplitudes collected in the baseline I/O function to a repeated-measures ANOVA with one between-subjects factor (dose) and one within-subjects factor (stimulus intensity). When significant interactions were found between dose and intensity, planned comparisons were performed to contrast each dose group with controls and evaluated using the Holm-Bonferroni correction to control for the number of comparisons. Paired pulse functions were evaluated with repeated-measures ANOVA with one between-subjects factor (dose) and two within-subjects factors (interval and intensity). In the event of significant interactions, stepdown ANOVA was performed, collapsing across intensity or IPI where appropriate. When significant effects of dose were obtained, mean contrast tests were conducted using Dunnett’s *t* statistic. One-tailed tests for multiple comparisons of tests of excitatory and inhibitory synaptic transmission were employed based on predictions of diminished synaptic responsiveness, as observed in previous work with low-level thyroid hormone insufficiency and hippocampal physiology (Gilbert 2004; Gilbert and Paczkowski 2003; Gilbert and Sui 2006; Sui and Gilbert 2003).

## Results

### General estimates of toxicity

**Figure 1:**
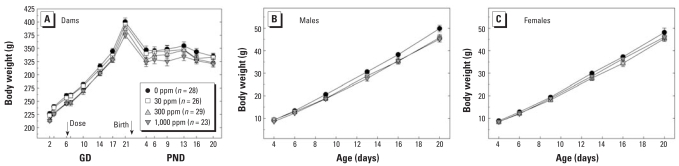
Body weights (mean ± SE) in dams (*A*) and male (*B*) and female (*C*) pups exposed to perchlorate.

We found no evidence of maternal toxicity in dams exposed to perchlorate (Figure 1A). Animals gained weight during pregnancy at the same rate, and body weights after parturition were not different among the groups. We observed a significant main effect of day [*F*(13, 1,326) = 706.89; *p* < 0.0001] but no significant effects of dose [*F*(3, 102) = 2.31; *p* > 0.08] or dose × day interaction [F(39, 1,326) = 0.54, *p* > 0.93].

Analysis of pup body weights (Figure 1B,C) also revealed significant main effects of day [*F*(5, 475) = 3,429; *p* < 0.0001] and sex [*F*(1, 95) = 11.44; *p* < 0.0010], but no overall main effect of dose [*F*(3, 95) = 2.0; *p* > 0.12], dose × sex [*F*(3, 95) = 0.92; *p* > 0.43], or dose × day × sex interactions [*F*(15, 475) = 0.34; *p* > 0.99]. Although a significant dose × day interaction was revealed [*F*(15, 475) = 2.22; *p* < 0.005], mean contrasts tests failed to identify any significant reduction in pup body weight at any age in either sex.

Eye opening was not delayed, nor did brain or hippocampal weights differ across dose groups (Table 1; all *p*-values > 0.50). Water intake of the dam was comparable across dose groups, and intake of perchlorate estimated from water consumption values is summarized in Table 2.

### Thyroid hormone analyses

**Figure 2:**
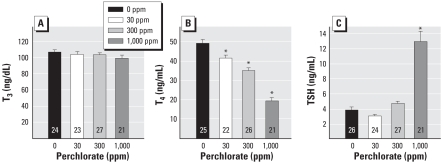
Thyroid hormone concentrations in dams (mean ± SE) treated with perchlorate beginning on GD6 and sacrificed on PND30. (*A*) T_3_. (*B*) T_4_. (*C*) TSH. Numbers within bars represent sample sizes. *p < 0.05 by Dunnett’s *t*.

Dams were sacrificed on the day pups were weaned (PND30), and blood was collected for hormone analysis. No changes in serum T_3_ levels were detected in the dam [*F*(3, 91) = 1.07; *p* > 0.36] (Figure 2A). Serum T_4_ levels in dams were reduced [*F*(3, 90) = 55.44; *p* < 0.0001] in a dose-dependent manner by 16%, 28%, and 60%, for 30-, 300-, and 1,000-ppm dose groups, respectively (Figure 2B). Dam TSH serum levels were increased 3-fold, and this increase was restricted to the highest dose level, as shown in Figure 2C [*F*(3, 94) = 45.41; *p* < 0.0001].

**Figure 3:**
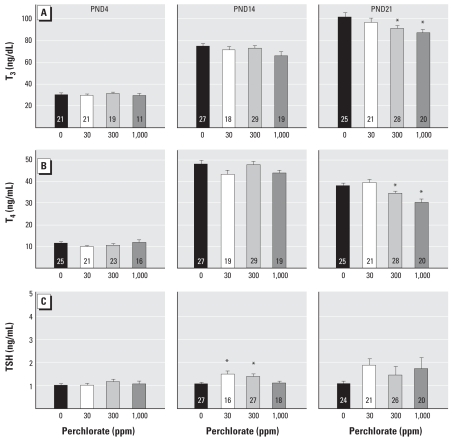
Thyroid hormone concentrations (mean ± SE) in pups exposed to perchlorate beginning on GD6 and sacrificed on PNDs 4, 14, or 21. (*A*) T_3_. (*B*) T_4_. (*C*) TSH. Data from PND4 represent males and females because samples were pooled to provide sufficient serum for the assays. No differences in serum hormones were detected between sexes on PND14 and PND21 (*p* > 0.05), so data were collapsed across, and mean value per litter at each age was analyzed. Numbers within the bars represent sample sizes. *p < 0.05 by Dunnett’s *t*.

Serum levels of thyroid hormone were determined in trunk blood sampled from pups on PNDs 4, 14, and 21. As minimal blood is available in young animals, samples were pooled from all culls within a litter, regardless of sex, on PND4. Individual serum samples from males and females at PNDs 14 and 21 were evaluated; no differences were seen between sexes (all *p*-values > 0.05), so a mean hormone value was calculated per litter. As shown in Figure 3A, we observed no differences in serum T_3_ concentrations in pups on PND4 [*F*(3, 68) = 0.39; *p* > 0.76] or PND14 [*F*(3, 89) = 2.19; *p* > 0.094], but a 10–14% reduction in T_3_ relative to control levels was observed on PND21 [*F*(3, 90) = 3.22; *p* < 0.0263]. Mean contrast tests indicated that this reduction in T_3_ was evident at the 300- and 1,000-ppm dose groups (Dunnett’s *t*, *p* < 0.05). No significant changes in serum T_4_ levels were detected in pups on PND4 [*F*(3, 81) = 1.40; *p* > 0.24] or PND14 [*F*(3, 90) = 1.94; *p* > 0.129]. A modest but significant reduction in T_4_ was seen in PND21 pups [*F*(3, 90) = 9.88; *p* < 0.0001] in the 300-and 1,000-ppm dose groups (~ 9–20% reduction relative to control levels, Dunnett’s *t*, *p* < 0.05). Serum TSH data from pups are summarized in Figure 3C. No change in TSH was detected in serum from PND4 animals [*F*(3, 58) = 0.58; *p* > 0.62]. TSH was elevated marginally in pups on PND14 [*F*(3, 82) = 4.82; *p* < 0.0039] and seen only at the intermediate dose levels (Figure 3C). Very modest increases in TSH were detected on PND21 but were more variable and failed to reach statistically significant levels [*F*(3, 87) = 1.23; *p* > 0.30]. All serum hormone concentrations had returned to control levels in adulthood.

### Behavioral assessments

We detected no group differences in any of the behavioral studies performed in perchlorate-treated animals. No difference between treatment groups was seen in either horizontal or vertical motor activity (Supplemental Material, Figure 1, available online at http://www.ehponline.org/members/2008/11089/suppl.pdf). Latency to find the hidden platform in the Morris water maze was reduced over days in all groups, indicating learning of the task, but no differential rate of acquisition was seen as a function of perchlorate treatment [Supplemental Material, Figure 2 (online at http://www.ehponline.org/members/2008/11089/suppl.pdf)]. Similarly, in probe trials, the percentage of time animals spent in the correct quadrant increased on successive trials in control and perchlorate-treated animals. Trace fear conditioning using a robust training paradigm of six CS–US pairings showed clear evidence of conditioning to cue in the 24 hr after the posttraining test session, but no differences among treatment groups were evident [Supplemental Material, Figure 3A (online at http://www.ehponline.org/members/2008/11089/suppl.pdf)]. Similarly, conditioning to context, evaluated by placing the animal back in the original test box 24 hr after training, did not differ among the groups [Supplemental Material, Figure 3B (online at http://www.ehponline.org/members/2008/11089/suppl.pdf)].

### Electrophysiologic assessments

Examination of histologic material resulted in elimination of a total of 12 animals because of inaccurate electrode placement, and these deletions were equally distributed across dose groups. Supplemental Material, Figure 4 (online at http://www.ehponline.org/members/2008/11089/suppl.pdf) depicts electrode placements in the animals included in the analyses below.

### Tests of excitatory synaptic transmission

**Figure 4:**
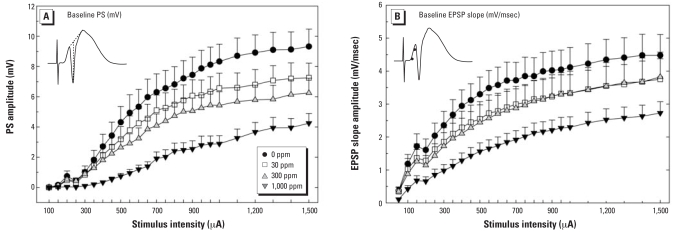
Amplitudes (mean ± SE) of PS (*A*) and EPSP slope (*B*) in the dentate gyrus in adult male rats exposed to perchlorate from GD6 to PND30. The inset in (*A*) depicts a typical response to a high-intensity pulse and how the PS was scored on each averaged waveform at each intensity for each subject. The inset in (*B*) depicts a response to a high-intensity pulse and the points on the rising phase of the waveform between which the slope was calculated as an estimate of EPSP amplitude.

I/O functions describe the relationship between intensity of the applied current and magnitude of the resulting evoked synaptic response. Changes in slope of the curve describing this relationship indicate alterations in excitatory synaptic transmission at the perforant path to dentate granule cell synapse. Persistent reductions in the efficacy of excitatory synaptic transmission were induced by developmental exposure to perchlorate in adult male offspring. Figure 4 displays the group mean absolute values for PS (millivolts of negative component of the field potential as shown in the inset of Figure 4A) and EPSP slope amplitude (millivolts per millisecond; slope of line between two points is shown in the inset of Figure 4B) recorded in the dentate gyrus of control and perchlorate-exposed animals. Clear dose-dependent reductions in amplitude of both measures are evident. The overall ANOVA revealed a significant main effect of dose [*F*(3, 60) = 4.38; *p* < 0.0075] and a significant dose × intensity interaction [*F*(72, 1,440) = 3.56, *p* < 0.0001] for the PS measure. Planned contrasts of the intensity response function using Holm-Bonferroni correction to control for type I error rate confirmed a significant reduction in PS amplitude at all dose levels tested: 0- versus 30-ppm [*F*(24, 1,440) = 1.51; *p* < 0.03], 0- versus 300-ppm [*F*(24, 1,440) = 3.83; *p* < 0.0001], and 0- versus 1,000-ppm dose groups [*F*(24, 1,440) = 9.82; *p* < 0.0001]. EPSP slope I/O functions followed a similar pattern, but the dynamic range of this measure is much smaller than for PS, and the data were more variable. The overall ANOVA failed to detect a statistically significant effect of dose [*F*(3, 60) = 1.74; *p* > 0.16] or a dose × intensity interaction [*F*(72, 1,440) = 1.14; *p* > 0.20], despite a clear reduction in amplitude evident at the high dose level (Figure 4B). Although no significant interaction was seen in the main analysis, planned contrasts of the intensity response function confirmed a significant dose × intensity interaction

### Tests of inhibitory synaptic transmission

**Figure 5:**
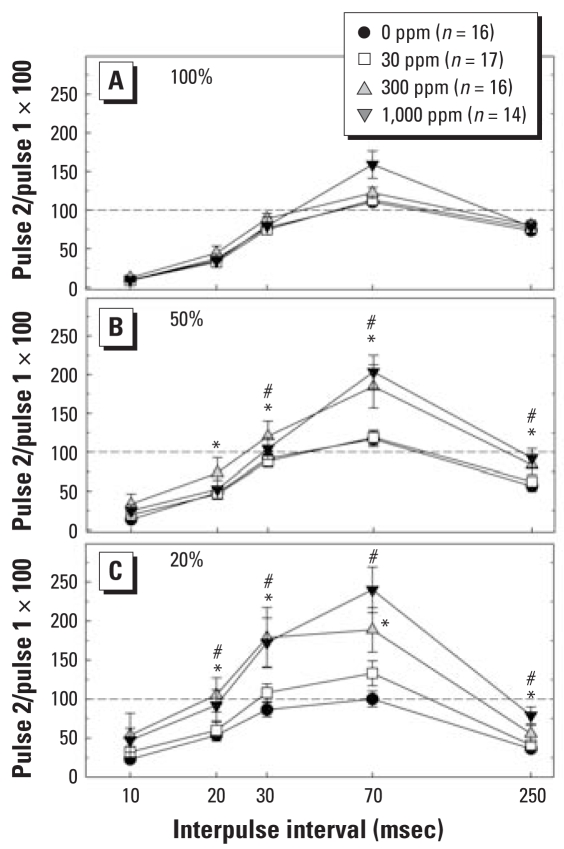
Paired pulse ratios (mean ± SE) across five IPIs in response to three stimulus intensities in adult male rats exposed to perchlorate from GD6 to PND30. (*A*) Maximal stimulus intensity. (*B*) Intensities producing PSs equivalent to 50% maximal. (*C*) Intensities producing PS equivalent to 20% of maximal. *p < 0.05 for 300 ppm compared with 0 ppm, and #*p* < 0.05 for 1,000 ppm compared with 0 ppm, by Dunnett’s *t*. limited to the 0- versus 1,000-ppm dose group [*F*(24, 1,440) = 4.10; *p* < 0.0001].

Paired pulse functions provide an index of the relative balance between excitatory and inhibitory influences on hippocampal output. Previous work in animals with diminished thyroid capacity induced by propylthiouracil (PTU) has revealed evidence of a shift in this balance toward a diminished inhibitory tone (Gilbert et al. 2006). A similar pattern was seen as a result of developmental perchlorate exposure. Results presented in Figure 5 demonstrate the time- (IPI) and intensity-dependent triphasic pattern of depression, facilitation, and depression in control animals. Strong inhibition produced by delivery of stimuli at maximal stimulus strength (100% maximum) revealed little difference among the groups (Figure 5A). A progressively increasing degree of dispersion is evident as conditioning pulse intensity is reduced to yield PS responses of 50% and 20% of maximal (Figure 5B,C). An upward shift in the entire paired pulse curve occurs as conditioning pulse intensity is reduced, and this shift is most dramatic in perchlorate-exposed animals at the lowest stimulus strength (Figure 5C). An overall three-way ANOVA yielded significant main effects of dose [*F*(3, 57) = 5.67; *p* < 0.0018], intensity [*F*(2, 114) = 21.51; *p* < 0.0001], and IPI [*F*(4, 228) = 130.39; *p* < 0.0001]. Significant dose × intensity [*F*(6, 114) = 4.46; *p* < 0.0004] and dose × IPI [*F*(12, 228) = 3.30; *p* < 0.0002] interactions were also detected. Stepdown ANOVA to further explore the effects of intensity and IPI revealed that the alteration in synaptic inhibition occurred at the 20% [*F*(3, 301) = 10.17; *p* < 0.0001] and 50% [*F*(3, 311) = 5.59; *p* < 0.0010] stimulus intensities. Significant differences at the 300- and 1,000-ppm dose levels were detected at both stimulus intensity levels (Dunnett’s *t*, *p* < 0.05). Stepdown analysis of the main effect of IPI indicated that significant dose-related differences were evident at several IPIs. No differences were seen at the shortest IPI of 10 msec (*p* > 0.18). A significant effect was detected at the 20-msec IPI [*F*(3, 183) = 3.30; *p* < 0.0215], and Dunnett’s *t* indicated that this difference was limited to the difference between controls and the 300-ppm dose group. At IPIs of 30 msec [*F*(3, 183) = 4.22; *p* < 0.0065], 70 msec [*F*(3, 183) = 15.77; *p* < 0.0001], and 250 msec [*F*(3, 183) = 6.72; *p* < 0.0003], significant differences were seen in both the 300- and 1,000-ppm dose groups (Dunnett’s *t*, *p* < 0.05).

### Tests of synaptic plasticity

**Figure 6:**
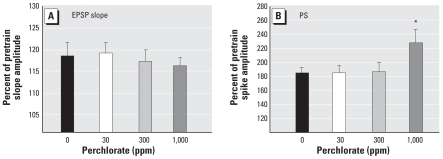
LTP (mean ± SE) of EPSP slope amplitude (*A*) and PS amplitude (*B*) in adult male rats exposed to perchlorate from GD6 to PND30. (*A*) Probe stimuli recorded before and after LTP-inducing trains of maximal stimulus strength (1,500 μA) increased EPSP slope amplitude, indicating that LTP was successfully induced in all groups to the same degree (*p* > 0.05). (*B*) LTP was also induced in the PS, but the magnitude of increase was greater in perchlorate-treated animals from the high-dose group. Note difference in scale between (*A*) and (*B*). *p < 0.05 by Dunnett’s *t*.

LTP is a well-established model of synaptic plasticity believed to embody the cellular substrate of learning and memory. Application of high-intensity trains produced significant increases in EPSP slope amplitude (~ 20% increase above pretrain baseline amplitudes), and the magnitude of this increase was comparable between control and perchlorate-treated animals [Figure 6A *F*,(3, 54) = 0.20; *p* > 0.85]. LTP-induced increases in PS amplitudes are of larger magnitude (~ 80% in controls) and greater increases were seen in the high dose of perchlorate than in controls (Figure 6B). ANOVA revealed a significant main effect of dose [*F*(3, 51) = 3.12; *p* < 0.0337], and mean contrast tests supported the conclusion of an augmented PS LTP in the 1,000-ppm perchlorate group (Dunnett’s *t*, *p* < 0.05).

## Discussion

Developmental exposure to perchlorate produced modest reductions in circulating thyroid hormones in dams and pups and no signs of overt toxicity. We detected no evidence of behavioral alterations in tests of motor activity, spatial learning, or fear conditioning. However, reductions in excitatory and inhibitory synaptic transmission were observed in the dentate gyrus of adult offspring of perchlorate-treated dams. These findings are the first to demonstrate that mild degrees of hormone insufficiency induced by perchlorate early in life lead to persistent deficits in synaptic function in adulthood. The absence of effect on behavioral assessments of learning and memory may be a function of dose, degree of hormonal disruption, duration of prenatal exposure, or the cognitive demands and sensitivity of the behavioral tasks.

### Perchlorate impairs hippocampal excitatory synaptic transmission

Perchlorate produced dose-dependent reductions in excitatory synaptic transmission in the dentate gyrus. Both EPSP and PS components of the compound field potential were decreased in amplitude by developmental exposure to perchlorate. These observations are consistent with findings of graded levels of thyroid hormone insufficiency induced by PTU (Gilbert and Sui 2006). The EPSP slope measure is the summed synaptic activation at the level of the dendrites where the bulk of synaptic connections are made between cortical afferents and hippocampal neurons. Reductions in field potential estimates of EPSP slope indicate reductions in afferent input, transmitter release, or postsynaptic responsiveness. Diminution of the somatic response, the PS, reflects a reduction in excitability of the cell. This could result from reduced cortical innervation, reduced synaptic responsiveness, or alterations in the firing properties of the granule cells. Collectively, these observations indicate a dose-dependent decline in the efficiency of synaptic transmission at this first synaptic junction into the hippocampal formation. Importantly, reductions in PS measures were evident at the lowest dose of perchlorate tested (30 ppm, 4.5 mg/kg/day).

### Perchlorate alters excitatory/inhibitory balance in dentate gyrus

In hippocampal circuits, activation of inhibitory interneurons dampens the firing of granule cells (PS) without affecting the synaptic component of the field response (EPSP slope). A standard test of inhibitory function delivers pairs of stimulus pulses close together in time—the degree of inhibition is reflected as a reduction in the amplitude of the PS of the second response relative to the first, and the magnitude of the suppression is stimulus dependent (Burdette and Gilbert 1995). A triphasic pattern of depression, facilitation, and depression is characteristic of paired pulse responses in the dentate gyrus and results from the influence of temporally successive and overlapping phenomena including recurrent inhibition, presynaptic facilitation, and feedforward inhibition.

Perchlorate produced a shift toward reduced depression/enhanced facilitation in paired pulse tests. Depression of the PS is most robust at very short intervals (early paired pulse depression). It is mediated by GABAergic interneurons synapsing on the soma of granule cells that serve to limit the degree of granule cell firing through feedback circuits (Fukuda et al. 1996; Papatheodoropoulos and Kostopoulos 1998). Feedback inhibition at the 10-msec IPI was not affected by perinatal perchlorate exposure at any dose or any intensity. However, a slight and significant reduction was evident at the 20-msec IPI at low to moderate intensities. As the interval between pulses is increased, this strong inhibition wanes and paired pulse facilitation predominates (IPIs of 50–70 msec). This interval corresponds to a time when increases in presynaptic transmitter release contribute maximally to the amplitude of the field potential amplitude generated by the second pulse of the pair. Facilitation is the summed effect of presynaptic and postsynaptic factors at the granule cell synapse and at the interneuron (Papatheodoropoulos and Kostopoulos 1998). The magnitude of facilitation was significantly enhanced in perchlorate-exposed animals at the lower stimulus intensities. A second period of depression follows facilitation and is induced with longer IPIs (250 msec). It is smaller in amplitude than early paired pulse depression and its mechanism is less well understood. Late paired pulse depression involves feedforward inhibitory circuits and can be modulated by antagonists of *N*-methyl-d-aspartate glutamate receptor (Gilbert and Burdette 1996; Joy and Albertson 1993). This form of paired pulse depression was also reduced by perchlorate at the 300-and 1,000-ppm dose levels.

The overall pattern of effects seen in tests of paired pulse inhibition is similar to that observed in the PTU model of hypothyroidism (Gilbert et al. 2006; Sui and Gilbert 2003). Reduced paired pulse inhibition may reflect a direct impact of hypothyroxenemia on phenotypic expression of inhibitory neurons, as previously described for PTU and methimazole (Berbel et al. 1996; Gilbert et al. 2006). In these studies, a reduction in expression of parvalbumin-containing interneurons was seen in hippocampus and cortex of hormone-deficient animals and was coupled with impairments in inhibitory synaptic transmission. Alternatively, reduced paired pulse inhibition may be a further manifestation of impaired glutamatergic activation at inhibitory synapses or of reduced release of GABA from interneuronal populations.

Whatever the mechanisms, interneuronal networks play a central role in generating behaviorally relevant network-driven patterns of activity in the adult brain. In the developing brain, even minor disturbances in this population of neurons alters the balance of excitation and inhibition and disrupts the fine-tuning of networks that determine the quality of information processing across cortical domains. Childhood epilepsy, schizophrenia, and autism have all been linked to a disruption in the development of populations of interneurons (Ben-Ari et al. 2004; Levitt et al. 2004). A relative state of disinhibition may manifest as increased susceptibility to seizures, a speculation consistent with recent reports in animals experiencing brief and transient episodes of thyroid hormone insufficiency *in utero* (Auso et al. 2004). A reduction in inhibitory function may also contribute to the augmentation of PS LTP in the dentate gyrus of thyroid hormone–compromised animals previously described for PTU (Gilbert 2004; Gilbert and Packzowski 2003; Gilbert and Sui 2006) and now for perchlorate.

### Perchlorate produces modest alterations in long-term synaptic plasticity

LTP of the EPSP slope was not altered in perchlorate-treated animals, a finding distinct from previous work with PTU. These data indicate that the mechanisms controlling synaptic plasticity are intact and distinct from those subserving baseline synaptic transmission. Paradoxically, yet consistent with the effects of PTU, a significant augmentation of PS LTP over control levels was observed (Gilbert 2004; Gilbert and Paczkowski 2003; Sui et al. 2005). Reductions in the tonic level of inhibition, as described above in paired pulse tests, may contribute to an augmentation of the PS amplitude after LTP induction. Alternatively, augmented PS LTP may derive from an increase in the synchrony of cell firing. Subtle changes in the structure of dendritic spines have been reported in hypothyroidism (Madiera et al. 1992) and can influence the efficiency of synaptic transmission and augment cell excitability (Carlisle and Kennedy 2005). Restructuring of spines triggered by LTP-like synaptic activation may represent a means whereby granule cells compensate for reduced synaptic input.

### Perchlorate and thyroid hormones: relationship to developmental neurotoxicity

Thyroid hormones were decreased in dams and pups as a function of perchlorate exposure, and reductions in synaptic transmission were observed at a dose that only marginally reduced (~ 15%) circulating levels of thyroid hormone in dams (30 ppm, 4.5 mg/kg/day). We are unaware of any nonthyroidal actions of perchlorate that could readily account for the present findings on hippocampal physiology. Previous published reports of the effects of perchlorate on neurodevelopment are limited to those of York et al. (2004, 2005a, 2005b), who summarized the results of findings from regulatory guideline studies. There was some degree of overlap in dose levels used in these studies and the present report (30 mg/kg/day, ~ 300 ppm; Table 1) and considerable overlap in serum thyroid hormone reductions. In the present study, 300-ppm and 1,000-ppm per-chlorate reduced T_4_ in pups by approximately 11% and 27%, respectively. The high dose of 30 mg/kg/day (~ 300 ppm) in the studies by York et al. (2005a) produced maximal effects on thyroid hormone reductions on PND21(~ 19% decreases from control values) and fell between our two high-dose groups.

At the time of adult testing, euthyroid conditions had returned, yet deficits in neurophysiologic measures persisted, indicating that a permanent change in brain function was induced as a consequence of altered thyroid status during development. Although modest reductions in serum hormones were seen in pups, no hormone estimates are available for the prenatal period. At weaning, serum T_4_ and TSH levels were more dramatically altered in dams than in pups. It is possible that prenatal hormone insufficiencies are critical for the observed changes in hippocampal circuitry. Although the granule cells of the dentate gyrus are of postnatal origin, the perforant path input to dentate gyrus is derived from axons of neurons in the entorhinal cortex, an area with a primarily prenatal developmental ontogeny. On the other hand, severe hypothyroidism beginning just before birth is sufficient to induce alterations in hippocampal structure and dentate physiology, and a recent report of selective transport and accumulation of perchlorate in the mammary gland suggests an increased vulnerability of the neonate to perchlorate (Anderson et al. 2003; Dohan et al. 2007; Gilbert 2004; Gilbert and Paczkowski 2003). Caution must be applied in attempts to directly link serum hormone concentrations and resulting brain dysfunction. Thyroid homeostasis is a dynamic, cyclical condition; the relationship of maternal, fetal, and neonatal serum hormone concentrations, windows of vulnerability, and differential tissue sensitivities during development are complex. Significant regulatory control over local intra-hippocampal concentrations of thyroid hormones occurs (e.g., brain deiodinases and transport proteins) such that brain hormone concentrations at the critical site or during the critical developmental window may not be reflected in serum hormone measures (Guadano-Ferraz et al. 1999; Visser et al. 2007). The pharmacokinetics and dosimetry of perchlorate, its relationship to iodine inhibition and thyroid hormone reductions during pregnancy and infancy, and the subsequent impact on brain development require further study.

### Developmental exposure to perchlorate: impact on adult behavior

The absence of effect of perchlorate on motor activity in the adult is consistent with previous reports (York et al. 2004, 2005a). In the present study, no effects of perchlorate were observed in trace fear conditioning. We had anticipated deficits because this form of fear conditioning has been shown to rely upon the activation and integrity of the hippocampus (Chowdhury et al. 2005). However, in animals with hippocampal lesions, Wiltgen et al. (2006) recently demonstrated impaired trace fear conditioning after a single CS–US pairing; this impairment was overcome with additional CS–US presentations. Robust conditioning induced by the multiple CS–US pairings in the present study may have masked subtle impairments in trace fear conditioning. York et al. (2004) did not detect learning deficits in adult offspring of perchlorate-treated dams using passive avoidance conditioning and a simple position discrimination task. In the present study, we used a more complex test of spatial learning, the Morris water maze, but also failed to uncover deficits in perchlorate-treated animals. The lack of effect on spatial learning was surprising given previous observations of altered hippocampal synaptic transmission coupled with spatial learning impairments after thyroid hormone disruption induced by PTU or methimazole (Akaike et al. 1991; Gilbert and Sui 2006). However, relative to perchlorate, the degree of hormone suppression induced by PTU and methimazole was more severe (Akaike et al. 1991; Gilbert and Sui 2006), and significant differences among these treatments exist in mechanism of toxicity, kinetics, and dosimetry.

The lack of effect of developmental perchlorate exposure on learning was also surprising in the context of such severe reductions in hippocampal synaptic transmission. However, to the extent that learning is reflected in tests of synaptic plasticity, the failure of perchlorate to detrimentally impact hippocampal LTP is consistent with a lack of effect on behavioral plasticity. It is possible that the augmentation of PS LTP is a reflection of an adaptive or compensatory response in cell physiology that aids in the reversal of learning deficits. Alternatively, many other brain regions are engaged in the performance of even simple learning tasks, and as such, the sluggish physiology of one region may not be mimicked in other brain regions or be directly reflected in global downstream measures. In addition, significant behavioral compensation may mask underlying behavioral deficits that are apparent earlier in development or may be revealed with more demanding cognitive tasks. Despite the lack of behavioral effects in the specific tasks used in the present study, impairment of synaptic transmission in adult offspring clearly indicates that a permanent detriment in brain function remains as a consequence of developmental perchlorate exposure.

## Conclusions

In summary, hypothyroxenemia induced in dams and pups by developmental exposure to perchlorate was associated with deficits in excitatory and inhibitory synaptic function in the hippocampus. Physiologic end points provide an integrated measure of the functional consequences of early disruption of the thyroid axis and its impact on brain structure and development. Effects were dose dependent, evident at mild levels of hormone insufficiency, and persisted despite return to normal thyroid status at the time of testing. Decrements in hippocampal synaptic LTP were not detected, nor were deficits in hippocampal-based learning tasks, perhaps related to the relative sparing of synaptic plasticity. Recent reports from the CDC demonstrating a strong association between perchlorate exposure and serum levels of thyroid hormone in women with marginal iodine deficiencies raise considerable public concern about the impact of this contaminant on the developing fetus (Blount et al. 2006). The present data provide evidence in a rodent model that modest degrees of thyroid hormone reduction induced by perchlorate result in persistent decrements in brain function.

## Figures and Tables

**Table 1:** Brain and hippocampal weights (mean ± SE) by dose group at PNDs 4, 14, and 21 in male and female offspring.

	Male				Female			
Dose (ppm)	0	30	300	1,000	0	30	300	1,000
Brain weight (g)								
PND4 (M + F)^a^	0.324 ± 0.003	0.321 ± 0.005	0.328 ± 0.003	0.311 ± 0.003				
PND14	1.139 ± 0.0138	1.126 ± 0.0268	1.149 ± 0.0129	1.100 ± 0.0233	1.114 ± 0.0146	1.096 ± 0.0221	1.104 ± 0.0218	1.106 ± 0.0158
PND21	1.404 ± 0.0171	1.374 ± 0.0311	1.394 ± 0.0129	1.383 ± 0.0276	1.350 ± 0.0146	1.369 ± 0.0171	1.353 ± 0.0196	1.352 ± 0.0242
Hippocampus weight (mg)								
PND14	35.0 ± 1.70	37.0 ± 1.61	36.5 ± 1.83	34.7 ± 2.27	34.9 ± 1.69	35.5 ± 1.72	35.7 ± 1.30	36.1 ± 1.09
PND21	50.2 ± 1.66	49.8 ± 2.15	47.8 ± 1.45	46.9 ± 1.97	46.6 ± 1.16	45.8 ± 2.37	46.6 ± 1.06	47.4 ± 1.37

Brain and hippocampal weights increased with age; no treatment-related effects of perchlorate exposure were noted (*p >* 0.05).

a Because of insufficient samples for each sex on PND4, mean brain weights were calculated per litter, pooling across sex.

**Table 2:** Water consumption and perchlorate intake of dams between GD6 and PND9.

Drinking water concentration (ppm)	Water consumption (mL/kg/day)	Perchlorate intake (mg/kg/day)	No. of dams with litter
0	138.3 ± 3.47	0	28
30	142.0 ± 4.43	4.5 ± 0.11	26
300	135.1 ± 2.91	44.2 ± 0.70	29
1,000	132.2 ± 3.40	140.3 ± 2.95	23

Perchlorate was calculated from water consumption and dam body weights.
